# The Interplay between Severe Cirrhosis and Heart: A Focus on Diastolic Dysfunction

**DOI:** 10.3390/jcm13185442

**Published:** 2024-09-13

**Authors:** Dragoș Lupu, Laurențiu Nedelcu, Diana Țînț

**Affiliations:** 1 Department of Fundamental, Prophylactic, and Clinical Disciplines, Transilvania University of Brasov, 500036 Brașov, Romania; dragos182@yahoo.com; 2 ICCO Clinics Brasov, Transilvania University of Brasov, 500059 Brașov, Romania; dianatint@gmail.com; 3 Department of Medical and Surgical Specialties, Transilvania University of Brasov, 500036 Brașov, Romania

**Keywords:** diastolic dysfunction, cirrhotic cardiomyopathy, echocardiography, hepatic cirrhosis

## Abstract

**Background/Objectives**: Cardiovascular involvement in severe cirrhosis presents diagnostic challenges and carries significant prognostic implications. This study aims to evaluate the relationship between liver disease severity and portal hypertension with the burden of diastolic dysfunction. **Methods**: We prospectively enrolled patients with hepatic cirrhosis, classified according to the Child–Pugh criteria. Of the 102 patients included, 65 were classified as Group A (non-severe cirrhosis: Child–Pugh Classes A and B) and 37 as Group B (severe cirrhosis: Child–Pugh Class C). Portal vein and spleen diameters were assessed using abdominal ultrasound. All patients underwent echocardiographic evaluation. LV systolic function was assessed by measuring ejection fraction, while diastolic function was evaluated using three parameters: E/Em ratio, E/Vp ratio, and indexed left atrial volume. **Results**: We observed a significantly greater burden of diastolic dysfunction in Group B compared to Group A. Specifically, the E/Vp ratio was 2.2 ± 0.4 in Group B versus 1.9 ± 0.3 in Group A (*p* < 0.001); the indexed LA volume was 34.5 ± 3.2 mL/m^2^ in Group B versus 30.1 ± 2.9 mL/m^2^ in Group A (*p* < 0.001); and the E/Em ratio was 17.0 ± 3.0 in Group B versus 11.5 ± 2.8 in Group A (*p* < 0.001). Additionally, the mean diameters of the portal vein and spleen were larger in Group B, with measurements of 14.3 ± 2.1 mm versus 11.5 ± 1.6 mm for the portal vein and 15.0 ± 1.2 mm versus 11.7 ± 1.5 mm for the spleen (*p* < 0.001), which correlated with the extent of diastolic dysfunction. **Conclusions**: Diastolic dysfunction was prevalent in 55% of patients with liver cirrhosis. The burden of diastolic dysfunction was higher in patients with severe hepatic cirrhosis compared to those with milder forms, and it correlated with the severity of portal hypertension, as assessed by measuring portal vein diameter and spleen diameter.

## 1. Introduction

Liver cirrhosis is a severe condition characterized by the progressive scarring of liver tissue. It can arise from various causes, including viral infections (such as hepatitis B and C), chronic alcohol consumption, obesity, and metabolic diseases. As the global prevalence of cirrhosis increases, it poses significant challenges not only to individual health, but also to healthcare systems, increasing the demand for treatments, hospitalizations, and other medical resources. Complications of cirrhosis include liver failure, gastrointestinal hemorrhages, heightened risk of liver cancer, and heart failure. Patients with liver cirrhosis exhibit well-documented hemodynamic alterations, including elevated cardiac output and reduced systemic vascular resistance [1,2]. The vasodilation that occurs in patients with cirrhosis can mask early systolic cardiac dysfunction and the initial decrease in contractility by reducing afterload and increasing preload, which in turn increases cardiac output. Resting diastolic evaluation may be inadequate for patients whose symptoms are limited to exertional dyspnea, as the increase in left ventricular filling pressures and pulmonary congestion in these individuals may only manifest during exercise [3]. Over the past three decades, research has highlighted a phenomenon known as “cirrhotic cardiomyopathy”, which includes impaired myocardial contractility, altered diastolic relaxation, and electrophysiological abnormalities in the absence of overt heart disease [4,5,6,7].

While the exact prevalence of cirrhotic cardiomyopathy remains unclear, estimates suggest that around 50% of cirrhotic patients may develop this type of cardiomyopathy at some stage in their illness [8]. Cirrhotic cardiomyopathy is often under-recognized in clinical practice, as it may only become apparent under stress, making diagnosis challenging.

The presence of cirrhotic cardiomyopathy, marked by left ventricular diastolic dysfunction, is associated with accelerated disease progression and a poorer prognosis. Increased stiffness of the cirrhotic heart can lead to reduced compliance, resulting in diastolic dysfunction. This condition can be assessed using transmitral Doppler echocardiography, tissue Doppler echocardiography, and cardiac magnetic resonance imaging. Additionally, there seems to be a correlation between diastolic dysfunction and the severity of liver dysfunction, as well as the presence of ascites [9].

In 2019, the Cirrhotic Cardiomyopathy Consortium redefined the diagnostic criteria for cirrhotic cardiomyopathy, incorporating the latest recommendations from the American Society of Echocardiography (ASE) and the European Association of Cardiovascular Imaging (EACVI) for assessing systolic and diastolic function. This update superseded the diagnostic recommendations from the 2005 World Congress of Gastroenterology [10]. Diastolic dysfunction is frequently observed in cirrhotic patients, with prevalence rates ranging from 43% to 70%, even when left ventricular ejection fraction (LVEF) remains normal [11].

The Consortium recommends using parameters such as the septal velocity of the Em wave, the E/Em ratio, indexed left atrial (LA) volume, and tricuspid regurgitation velocity for evaluating diastolic function.

While specific guidelines on whether the diastolic function should be assessed at rest or during exercise are lacking, it is crucial to recognize that diastolic abnormalities may not manifest at rest. Diastolic dysfunction symptoms often appear only during exercise, as left ventricular filling pressure may be normal at rest, but increases with physical activity due to the heart’s inability to increase cardiac output without increasing filling pressure [12].

Diastolic dysfunction has proven to be the most sensitive indicator for diagnosing cirrhotic cardiomyopathy, as it is the earliest parameter to be affected. Atroush and colleagues utilized tissue Doppler imaging and speckle tracking to evaluate diastolic function in patients with end-stage liver disease, investigating the correlation between cardiac dysfunction and the Child–Pugh classification of liver cell failure. The study identified a high prevalence of diastolic dysfunction (87.5%) among patients with end-stage liver disease by measuring the E/É ratio using tissue Doppler imaging (TDI), which was found to be more accurate than the E/A ratio. Although the study did not find a correlation between cardiac dysfunction and the severity of liver disease, it is important to note that it had a small sample size, consisting of only 40 patients who were followed for three months [13].

A meta-analysis by Stundiene et al., encompassing 16 studies, found that approximately 51% of cirrhotic patients have diastolic dysfunction [14]. However, these findings are limited by the use of less specific parameters, such as the E/A ratio and the inclusion of patients with potential ventricular relaxation impairment from other causes. As noted by Prekumar et al. and Ruíz-del-Árbol et al., inadequate detection of diastolic dysfunction may lead to adverse clinical outcomes. Therefore, early and accurate identification of this condition is crucial for improving patient prognosis [15,16].

The presence of cirrhotic cardiomyopathy and impaired diastolic function represent critical factors, especially for patients following liver transplantation. Ershoff et al. conducted a retrospective cohort study involving 254 liver transplant recipients to evaluate the impact of diastolic dysfunction on mortality. The LA volume index was used as a key parameter for assessment. The study concluded that this condition is associated with increased mortality in post-liver transplant patients, particularly those with a high Model for End-Stage Liver Disease (MELD) score [17]. Similarly, a 2022 study by Vetrugno et al., which included 83 orthotopic liver transplant recipients, explored the relationship between preoperative diastolic dysfunction and the risk of early allograft dysfunction. The study found that patients with impaired diastolic function were more likely to develop early allograft dysfunction after orthotopic liver transplantation [18].

This study aimed to analyze the correlation between the severity of liver disease (severe Child–Pugh C vs. non-severe Child–Pugh A and B) and the burden of diastolic dysfunction measured after exercise using a combination of parameters. Additionally, it sought to evaluate the potential correlation between diastolic dysfunction and the severity of portal hypertension, as assessed by abdominal ultrasonography parameters, specifically portal vein and spleen diameters.

## 2. Materials and Methods

We conducted an observational, prospective study involving 102 patients with hepatic cirrhosis, classified according to Child–Pugh criteria. Patient recruitment and subsequent clinical, laboratory, and abdominal ultrasound evaluations were conducted at the internal medicine clinic from November 2021 to March 2024. Within the first 15 days after inclusion, participants underwent cardiac ultrasound evaluations at a cardiology outpatient clinic. The inclusion and exclusion criteria are detailed in Table 1.

This study was approved by the Ethics Committee of Transilvania University of Brașov (approval number no.5, approval date: 26 February 2020), and all participants provided informed consent prior to enrollment.

Laboratory tests were performed to assess blood cell count, liver function (bilirubin, AST, ALT, GGT, LDH), renal function (creatinine, urea), coagulation status (INR), plasma albumin, and glucose levels. Additionally, the glomerular filtration rate (GFR) was calculated for all patients. Abdominal ultrasound evaluations focused on the dimensions of the liver, spleen, portal vein, and splenic vein, as well as the presence or absence of ascitic fluid.

The Child–Pugh score was assessed for each patient, following the classification system described in the MSD Manual (https://www.msdmanuals.com/professional/multimedia/clinical-calculator/child-pugh-classification-for-severity-of-liver-disease, accessed on: 30 July 2024). This score typically categorizes patients into three groups based on liver function severity: Class A (score of 5–6), Class B (score of 7–9), and Class C (score of 10–15). For our study, patients were divided into two groups: Group A (non-severe cirrhosis, encompassing Child–Pugh Classes A and B) and Group B (severe cirrhosis, Child–Pugh Class C).

Regarding cirrhosis etiology, the majority of patients had alcoholic cirrhosis (77 patients, representing 75% of the total). Eighteen patients had viral etiology (17%), while seven patients were classified under other etiologies (6%).

To assess systolic and diastolic function, all participants performed five squats and were subsequently evaluated via echocardiography.

Modern echocardiographic techniques are used to evaluate systolic and diastolic function for diagnosing cirrhotic cardiomyopathy. Left ventricular (LV) systolic function is commonly assessed by measuring the LV ejection fraction (LVEF), with values below 50% considered abnormal. For the assessment of diastolic function, four echocardiographic parameters may be used: septal and lateral mitral annular peak early diastolic velocity (e’), the ratio of the peak velocity of mitral inflow during early diastole (E) to the average of septal and lateral e’ (E/e’), LA volume indexed to body surface area, and tricuspid regurgitation velocity [3].

In our study, cardiac ultrasound was performed using a General Electric Vivid E9 machine with a 4.2 MHz cardiac transducer. A thorough examination was carried out, which included measurements of the dimensions of the aorta, left atrium, left ventricle, right atrium, right ventricle, interventricular septum, and posterior wall. The assessment also encompassed valve functionality, systolic pulmonary artery pressures, and evaluation of the pericardium. Systolic function was measured by calculating the LVEF using the Simpson method. Diastolic function was assessed using the following parameters:

**E/Em Ratio**: The ratio of E, the velocity of early mitral inflow measured by pulsed Doppler, to Em, the early diastolic mitral annular tissue velocity. Normal values are <15.

**E/Vp Ratio**: The ratio of E to Vp, where Vp represents the propagation of early diastolic trans-mitral velocity assessed by M-mode echocardiography. Normal values are <2, with values >2.5 linked to pulmonary capillary wedge pressures above 15 mmHg [19].

**Indexed Left Atrial (LA) Volume**: Normal values are <34 mL/m^2^.

Peak E wave velocity was evaluated in the apical four-chamber view using color flow imaging to achieve optimal alignment of PW Doppler with the mitral flow. We analyzed the peak modal velocity during late diastole (following the P wave on the ECG) at the leading edge of the spectral waveform.

Pulsed-wave tissue Doppler imaging (TDI) e’ velocity (cm/s) was measured in the four-chamber view using a pulsed-wave sample volume positioned at the lateral and septal basal regions, facilitating the calculation of the average e’ velocity. We assessed the peak modal velocity during early diastole at the leading edge of the spectral waveform.

For E/Vp acquisition, we used color M-mode in the apical four-chamber view, utilizing color flow imaging to guide M-mode cursor positioning. The color baseline was adjusted toward the mitral valve inflow to lower the velocity scale, enhancing the red/yellow inflow velocity profile. We then analyzed the slope of the inflow from the mitral valve plane into the left ventricular chamber during early diastole at a 4 cm distance.

To obtain the indexed LA volume, we used the apical four- and two-chamber views, capturing freeze frames before the mitral valve (MV) opening. The area–length method was utilized, with the left atrial appendage and pulmonary veins excluded from the tracings. The values were adjusted for body surface area.

**Figure 1:**
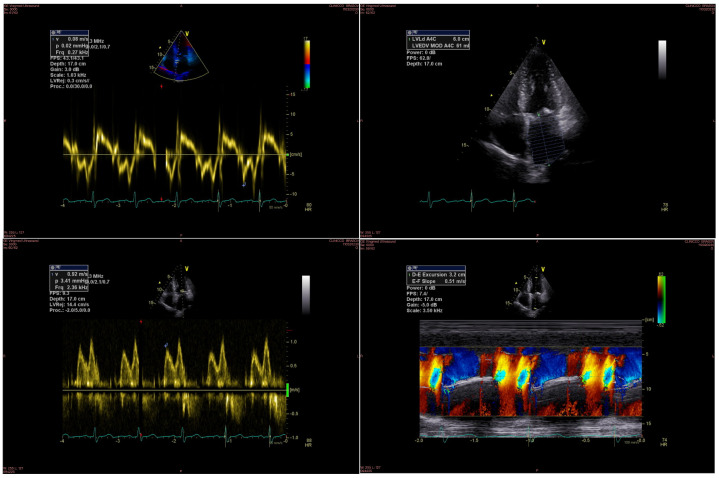
Diastolic function parameters measured by echocardiography: the upper left image shows the Em wave, the upper right image shows the LA volume, the bottom left image shows the E wave, and the bottom right image shows the Vp measurement.

An example of echocardiographic parameters used for evaluating diastolic function is displayed in Figure 1.

The physical exertion was supervised by medical staff, and echocardiography was performed immediately afterward. Following the evaluation, diastolic function parameters obtained from all three measurements were compared between the two study groups. Additionally, correlations were analyzed between diastolic function parameters and the diameters of the portal vein and spleen as measured with abdominal ultrasound.

### Statistical Analysis

Statistical analyses were conducted using Python 3.4 (PSF, Wilmington, DE, USA). Variables were reported as mean ± SD. Gender, smoking status, hypertension, dyslipidemia, and diabetes mellitus were recorded as dichotomous variables. The Chi-squared test was employed to compare the frequency of nominal variables. For continuous variables, the Independent-Samples *t*-test was used to compare means, while the Mann–Whitney U-test analyzed mean rank differences for ordinal variables. A two-sided *p*-value of <0.05 was considered statistically significant.

## 3. Results

Out of the 102 study participants, sixty-five patients had non-severe cirrhosis (Child–Pugh Classes A and B), while thirty-seven patients had severe cirrhosis (Child–Pugh Class C). Factors influencing diastolic function were similarly prevalent in both groups, regardless of the severity of hepatic disease (Table 2). Baseline characteristics were comparable between the two groups, with no significant differences in age, hypertension, dyslipidemia, coronary artery disease, or obesity. However, the prevalence of diabetes was notably higher in the severe cirrhosis group.

All patients were prescribed cardiovascular medication as shown in Table 3.

Significant differences were observed in medication prescriptions between patients with varying levels of hepatic function impairment. Beta-blockers were prescribed to all patients in the severe cirrhosis group, compared to only 46.2% in the non-severe group. Additionally, spironolactone and furosemide were more frequently administered in the severe group (91.9% vs. 3.1%, *p* < 0.001, and 86.5% vs. 3.1%, *p* < 0.001, respectively). In contrast, angiotensin-converting enzyme inhibitors were exclusively used in the non-severe group (24.6%). The dosage of furosemide ranged from 20 to 40 mg, while spironolactone was administered at doses between 25 and 50 mg. A total of ten patients were treated with SGLT2 inhibitors (dapagliflozin or empaglifozin), including four with non-severe cirrhosis and six with severe cirrhosis, all of whom had diabetes mellitus.

While LVEF was comparable between the two groups, significant differences were noted in diastolic function measurements between patients with non-severe and severe cirrhosis. Specifically, the E/Vp ratio was 1.9 ± 0.3 in the non-severe cirrhosis group versus 2.2 ± 0.4 in the severe cirrhosis group (*p* < 0.001); the indexed LA volume was 30.1 ± 2.9 mL/m^2^ compared to 34.5 ± 3.2 mL/m^2^ (*p* < 0.001); and the E/Em ratio was 11.5 ± 2.8 versus 17.0 ± 3.0 (*p* < 0.001).

Diastolic dysfunction was present in the majority of patients with severe cirrhosis, affecting 26 individuals (70%). Conversely, 34 patients in the non-severe group did not have this condition, leading to a lower prevalence in that group (47%). Overall, the prevalence of diastolic dysfunction across the entire study population was 55%.

Abdominal ultrasound parameters indicative of portal hypertension severity were markedly altered in patients with severe cirrhosis. The mean portal vein diameter was 14.3 ± 2.1 mm in the severe cirrhosis compared to 11.5 ± 1.6 mm in the non-severe cirrhosis group, group (*p* < 0.001). Additionally, the mean spleen diameter was significantly greater in the severe cirrhosis group (15.0 ± 1.2 mm vs. 11.7 ± 1.5 mm; *p* < 0.001), as shown in Table 4.

**Figure 2:**
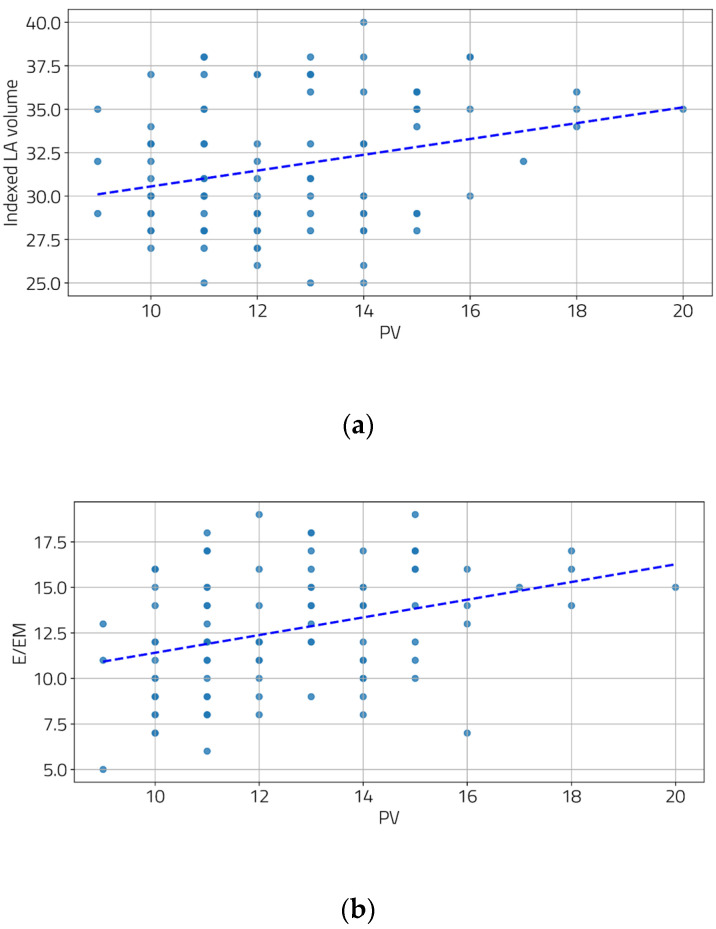
Correlation between PV and indexed LA volume (**a**) and E/EM (**b**). [(**a**) r = 0.28, *p* = 0.004; (**b**) r = 0.34, *p* < 0.001].

**Figure 3:**
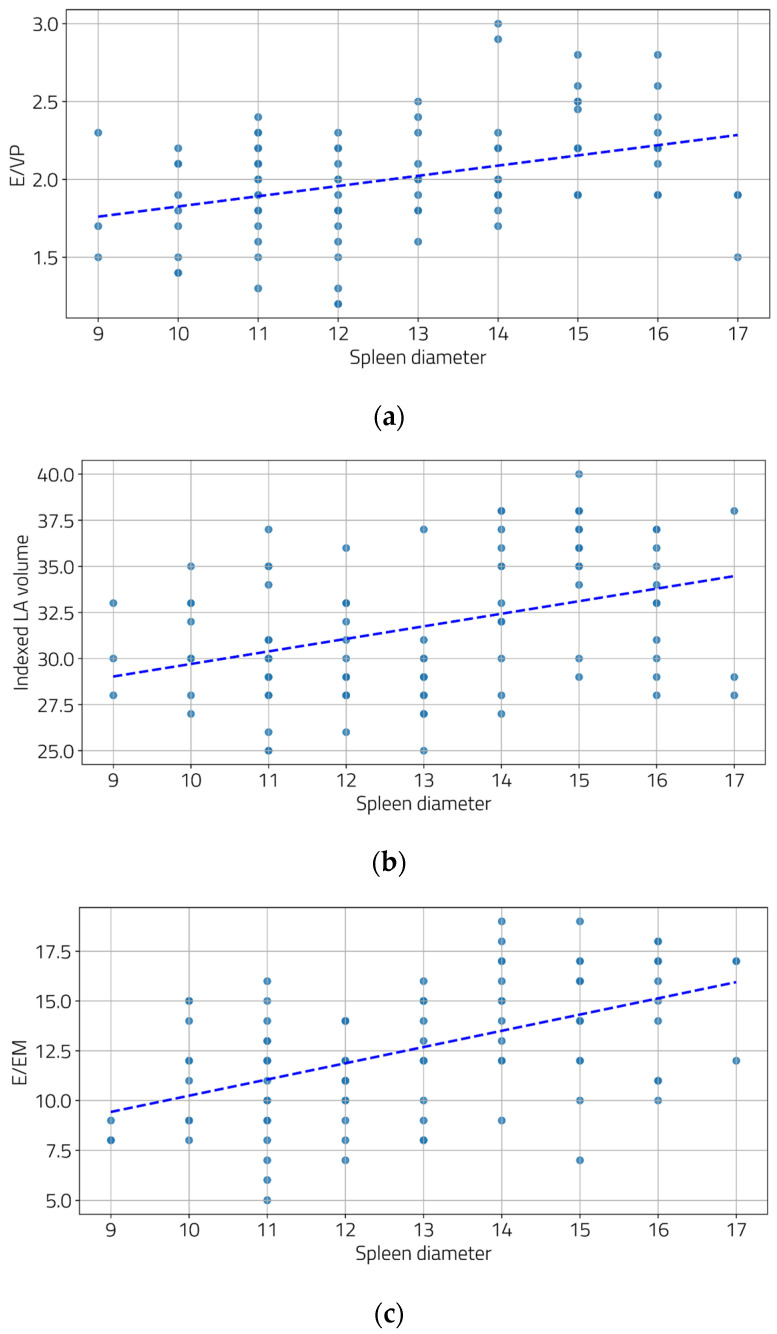
Correlations between spleen diameters and E/Vp (**a**), indexed LA volume (**b**), and E/Em (**c**). ((**a**) r = 0.39, *p* < 0.001; (**b**) r = 0.39, *p* < 0.001, and (**c**) r = 0.53, *p* < 0.001).

We also examined the relationship between the severity of portal hypertension and LV diastolic function. Moderate and statistically significant correlations were found between the portal vein diameter and two diastolic function parameters (Figure 2a,b), as well as between the spleen diameter and all three diastolic function parameters (Figure 3a–c).

## 4. Discussion

The clinical significance of diastolic dysfunction has gained considerable attention in recent years, with substantial evidence demonstrating its profound impact on both the quality of life and the prognosis of affected patients [20,21,22]. A meta-analysis by Ladeiras-Lopes et al. revealed that impaired diastolic function is associated with a 3.53-fold increased risk of cardiovascular events or death [23]. Similarly, research by Kosmala et al. indicated that asymptomatic LV diastolic dysfunction in patients with preserved systolic function is linked to the development of heart failure and decreased survival [24].

These findings underscore the importance of early detection and accurate assessment of diastolic dysfunction, particularly in patients with hepatic cirrhosis. Proper evaluation is crucial as it can significantly influence the prognosis of patients with impaired diastolic function, even when systolic function appears normal.

However, the presence and implications of diastolic dysfunction in patients with normal systolic function within the context of hepatic cirrhosis are less well-explored. Current studies are often limited by small sample sizes and inconsistent results, highlighting the need for further research in this area. Table 5 below summarizes the key studies on diastolic dysfunction in cirrhotic patients.

Our study conducted a comprehensive evaluation of diastolic function by incorporating three advanced parameters, including two (E/Em and indexed LA volume) recommended by the Cirrhotic Cardiomyopathy Consortium. Previous research has identified E/Em as a highly specific marker for elevated left ventricular filling pressure, while indexed LA volume serves as an indicator of chronically increased end-diastolic LV pressure, though it does not strongly correlate with LA pressure [31]. The third parameter, E/Vp, also proved to be valuable in assessing diastolic function [32]. Garcia et al. demonstrated that E/Vp is less dependent on left ventricular filling conditions; by combining Vp (an index of ventricular relaxation) with the E wave velocity (reflecting left atrial pressure), E/Vp is particularly useful for estimating left ventricular filling pressures [33]. To the best of our knowledge, our research is the first to utilize these three parameters to define diastolic dysfunction in cirrhotic patients.

Our findings revealed no significant differences in LV systolic function between the two groups, which aligns with the existing literature [34,35]. However, all three parameters used to define diastolic function were significantly more altered in patients with advanced hepatic disease, partially in line with findings from other studies [25,26,27,28]. While some previous studies have shown a link between diastolic dysfunction and the severity of cirrhotic disease, others, such as the research by Merli et al., found no such association [29,30].

By combining these three parameters, we were able to highlight the pressure changes in all three components: left ventricle, left atrium, and pulmonary capillary, reflecting the progression of these cardiac changes in parallel with the worsening of liver disease. Moreover, to support this hypothesis, we were able to show the correlation between the severity of portal hypertension and diastolic dysfunction. In our research, the degree of diastolic dysfunction correlates with both the portal vein diameter and spleen diameter.

Similar results were published in the literature. Thus, in a recent study, Behera et al. found that Child C patients with portal hypertension had a higher prevalence of cirrhotic cardiomyopathy and diastolic dysfunction [36]. Also, Marconi et al. concluded in their 2017 study that diastolic dysfunction, measured by Em and LA volume, worsens as portal pressures increase [37].

Since the focus of this research was on diastolic dysfunction, systolic function was assessed solely through the LVEF determined by the Simpson method, which revealed no statistically significant differences between the two groups. It is important to note that previous studies have demonstrated that global longitudinal strain (GLS) [38] and S wave velocity measured with tissue Doppler [39] are more effective methods for evaluating systolic function compared to ejection fraction.

Our study uniquely utilizes three distinct high-fidelity parameters: E/Vp, E/Em, and indexed LA volume to estimate diastolic function, correlating these measures with the severity of liver disease and assessing diastolic dysfunction after brief exercise. This approach allows us to uncover diastolic abnormalities that may not be evident under resting conditions. Furthermore, E/Vp has not been previously utilized as a marker for diastolic dysfunction in the context of hepatic cirrhosis.

Given that liver cirrhosis is associated with comorbidities such as malnutrition, muscle mass loss, reduced exercise capacity, and decreased muscle strength, we opted to use a non-standardized exercise test. This involved performing five squats, immediately followed by an assessment of diastolic function parameters.

To the best of our knowledge, this is the first study to utilize these three parameters to define diastolic dysfunction and to analyze them after exercise in patients with cirrhosis.

This study involved interdisciplinary collaboration, with patient enrollment conducted at an internal medicine clinic after a thorough evaluation and diagnosis. Cardiac assessments were performed separately in an outpatient cardiology clinic. Additionally, we implemented a novel approach by categorizing patients according to the severity of their cirrhosis—severe versus non-severe.

Our findings indicate that diastolic dysfunction, as a marker of heart failure, warrants consideration of specific interventions, particularly for patients with severe cirrhosis who have limited treatment options [40]. Thus, early recognition of diastolic dysfunction as a sign of heart failure (in conjunction with natriuretic peptides) could expand the therapeutic options by incorporating SGLT2 inhibitors into the treatment regimen. These drugs are now included in the guidelines with a Class I recommendation for the entire spectrum of heart failure, including heart failure with preserved LVEF [41]. Furthermore, earlier integration into a palliative care program should be considered, as it offers significant benefits for these patients [42].

The diagnosis of diastolic dysfunction is also critically important in post-liver transplant patients, as numerous studies have demonstrated an association with increased morbidity and mortality [43,44].

## 5. Conclusions

In our study, diastolic dysfunction was less prevalent in patients with non-severe cirrhosis, while the majority of patients with severe cirrhosis exhibited this condition. The overall prevalence of diastolic dysfunction among patients with liver cirrhosis was found to be 55%. Severe hepatic cirrhosis (Child–Pugh Class C) was also associated with a more pronounced degree of diastolic dysfunction compared to milder forms of the disease. This dysfunction was significantly correlated with the severity of portal hypertension, as reflected by surrogate parameters such as portal vein and spleen diameters. Spleen diameter was the sole factor that demonstrated a significant correlation with all three evaluated parameters of diastolic function. These findings underscore the importance of monitoring diastolic function in cirrhotic patients, particularly those with advanced disease, as early identification and management of diastolic dysfunction could potentially improve clinical outcomes.

### 5.1. Study Limitations

Our study has several limitations. The relatively small sample size may have impacted the thoroughness and significance of the results. Additionally, follow-up visits could have offered better insights into symptom progression and the development of diastolic dysfunction. Furthermore, measuring natriuretic peptides and examining their correlation with diastolic function and the severity of cirrhosis, along with the use of tissue Doppler ultrasound for GLS or S wave analysis, could significantly improve our understanding of these conditions.

### 5.2. Future Directions

Recognizing the importance of the early detection of diastolic dysfunction in reducing cardiovascular events after liver transplantation, a paradigm shift is essential. This shift involves incorporating diastolic function evaluated through modern diagnostic parameters into pre-liver transplant risk stratification, which has traditionally focused on ejection fraction and right-sided cardiac pressures.

## Figures and Tables

**Table 1:** Inclusion and exclusion criteria.

Inclusion Criteria	Exclusion Criteria
Age over 18 years	Age under 18 years
Diagnosis of hepatic cirrhosis Written informed consent	Heart failure from any cause other than CCM
	Persistent or permanent atrial fibrillation
	Significant ventricular arrhythmia
	Uncontrolled hypertension
	Acute coronary syndrome
	Ch Chronic kidney disease in hemodialysis stage Sequelae of ischemic or hemorrhagic stroke Inability to perform physical exertion

CCM = cirrhotic cardiomyopathy.

**Table 2:** Characteristics and comorbidities of the groups with non-severe cirrhosis compared to the severe cirrhosis group.

Characteristic	Non-Severe Cirrhosis n = 65	Severe Cirrhosis n = 37	*p*
Age, mean ± SD [years]	65.5 ± 10.9	64.5 ± 11.6	0.69
Male, n (%)	44 (67.7)	22 (59.5)	0.68
Hypertension Diabetes mellitus Chronic coronary syndrome Dyslipidemia, n (%)	32 (49) 8 (12) 5 (7) 12 (18)	17 (45) 11 (29) 4 (10) 9 (23)	0.74 **0.03** 0.45 0.34
Smoking, n (%)	23 (34.3)	15 (40.5)	0.61
BMI, mean ± SD [kg/m^2^]	24.2 ± 3.9	23.8 ± 4.5	0.84

BMI = Body mass index; GFR = estimated Glomerular Filtration Ratio; SD = standard deviation. Bold: statistical significance.

**Table 3:** Cardiovascular medication.

Medication	Non-Severe Cirrhosis n = 65	Severe Cirrhosis n = 37	*p*
Beta-blockers, n (%)	30 (46.2)	37 (100)	**<0.001**
Spironolactone, n (%)	2 (3.1)	34 (91.9)	**<0.001**
Furosemide, n (%) SGLT2 inhibitors, n (%)	22 (3.1) 4 (6)	32 (86.5) 6 (16)	**<0.001** **0.1003**
Angiotensin-converting enzyme inhibitors, n (%)	16 (24.6)	0 (0)	

SGLT2 = sodium-glucose transport protein 2. Bold: statistical significance.

**Table 4:** Comparison of cardiac and abdominal parameters assessed with ultrasound between the two patient groups.

Parameter	Non-Severe Cirrhosis n = 65	Severe Cirrhosis n = 37	*p*
LV Ejection fraction, mean ± SD [%]	56.9 ± 6.2	58.9 ± 3.9	0.06
E/VP, mean ± SD	1.9 ± 0.3	2.2 ± 0.4	<**0.001**
Indexed LA volume, mean ± SD [mL/m^2^]	30.1 ± 2.9	34.5 ± 3.2	<**0.001**
E/Em, mean ± SD	11.5 ± 2.8	17.0 ± 3.0	<**0.001**
PV, mean ± SD [mm]	11.5 ± 1.6	14.3 ± 2.1	<**0.001**
Spleen diameter, mean ± SD [mm]	11.7 ± 1.5	15.0 ± 1.2	<**0.001**

E = velocity of the early phase of mitral inflow; Em = early diastolic mitral annular tissue velocity LA = left atrium; LV = Left ventricle; PV = portal vein; VP = propagation of early diastolic trans-mitral velocity; SD = standard deviation. Bold: statistical significance.

**Table 5:** Main studies on diastolic dysfunction in cirrhotic patients.

Clinical Study	Number of Patients Enrolled	Aim	Results
A case-cohort study of left ventricular diastolic dysfunction in patients with cirrhosis: the liver–heart axis [25]	203	Assessment of the association of diastolic dysfunction with the factors affecting cirrhosis patients’ severity, complications, and survival	Higher Child–Pugh class, prolonged QTc, higher ascitic fluid protein levels, and poor survival are significantly associated with diastolic dysfunction
Cardiac dysfunction in cirrhotic portal hypertension with or without ascites [26]	60	To evaluate cardiac systolic and diastolic functions in liver cirrhosis patients with or without ascites	Diastolic dysfunction is commonly associated with advancement of hepatic dysfunction
Ultrasonographic Prevalence and Factors Predicting Left Ventricular Diastolic Dysfunction in Patients with Liver Cirrhosis: Is There a Correlation between the Grade of Diastolic Dysfunction and the Grade of Liver Disease? [27]	92	To assess the echocardiographic prevalence of diastolic dysfunction among a population of cirrhotic patients and to investigate whether a correlation between stage of cardiac dysfunction and stage of liver disease could be established	Diastolic dysfunction stage 1 is fairly prevalent among all CTP classes, whereas diastolic dysfunction stage 2 seems to be characteristic of the advanced liver disease (CTP-class C)
Diastolic myocardial dysfunction does not affect survival in patients with cirrhosis [28]	76	To investigate if diastolic dysfunction is associated with severity and etiology of cirrhosis and mortality	Diastolic dysfunction is more frequent in patients with ascites and does not link to mortality
Cardiac dysfunction in cirrhosis is not associated with the severity of liver disease [29]	74	To investigate factors associated with cardiac dysfunction in cirrhotic patients	No association between severity of liver disease and cardiac dysfunction
Systolic and diastolic impairment in cirrhotic cardiomyopathy: insights from a cross-sectional study [30]	68	To investigate the prevalence of systolic and diastolic function in patients with cirrhotic cardiomyopathy	Remarkable prevalence of cirrhotic cardiomyopathy Lack of correlation with the severity of liver cirrhosis

CTP = Child–Turcotte–Pugh, QTc—corrected QT interval.

## Data Availability

Dataset available on request from the authors.
